# The effect of music therapy on tinnitus: A systematic review

**DOI:** 10.1097/MD.0000000000036199

**Published:** 2023-12-15

**Authors:** Yunlian Niu, Yijie You

**Affiliations:** a Department of Neurology, Chongming Hospital Affiliated to Shanghai University of Medicine and Health Sciences, Shanghai, China; b Department of Neurosurgery, Chongming Hospital Affiliated to Shanghai University of Medicine and Health Sciences, Shanghai, China.

**Keywords:** chronic tinnitus, music therapy, standard music therapy, tinnitus, tinnitus loudness

## Abstract

**Objectives:**

Tinnitus is a common otological symptom affecting almost all aspects of life, especially the quality of daily life. The present study aims to analyze music therapy effect on tinnitus patients. This paper mainly analyzes 3 kinds of music therapy: Heidelberg model of music therapy (HMOMT), standard music therapy (SMT), and tailor-made notched music training (TMNMT). To provide a reference for the follow-up treatment of tinnitus, whether to take and what kind of music therapy.

**Method:**

A systematic literature search was performed in PubMed, Cochrane Library, EMBASE, Web of Science, and MEDLINE to obtain potential studies from their inception to May 2023 in all languages. Two researchers independently screened the studies, extracted data, and assessed the quality of the included studies. We included all randomized and non-randomized controlled trials that used music therapy to treat patients with tinnitus. We used fixed-effects and random-effect models to analyze data based on the heterogeneity results. The data analysis was performed by using Stata 12.0.

**Results:**

A total of 19 studies with 904 cases were included. Compared with before treatment, music therapy significantly reduces the tinnitus questionnaire score and tinnitus handicap inventory score. HMOMT, SMT, and TMNMT all significantly decrease tinnitus scores. Although the order of effectiveness of the 3 drugs is TMNMT > SMT > HMOMT, there is no statistical significance (*P* > .5).

**Conclusion:**

This meta-analysis of accumulated clinical trial data suggests that music therapy can relieve tinnitus symptoms and loudness. Among music therapies, SMT is recommended first for tinnitus based on cost, efficacy, and convenience. At the same time, TMNMT and HMOMT can be used as alternative therapies for specific cases.

## 1. Introduction

Tinnitus, a common otological symptom, is conscious awareness of a sound without an external auditory stimulus.^[1,2]^ Although the incidence of tinnitus varies between countries, the general trend has increased over time.^[3]^ It is associated with developments in modern society, including substance abuse, population aging, heavy work pressure, the rise of chronic diseases, and noise pollution.^[4]^ Tinnitus can affect almost all aspects of life, especially the quality of daily life. Some patients even suffer from emotional discomforts such as anxiety, depression, and insomnia.^[5,6]^

**Figure 1.**
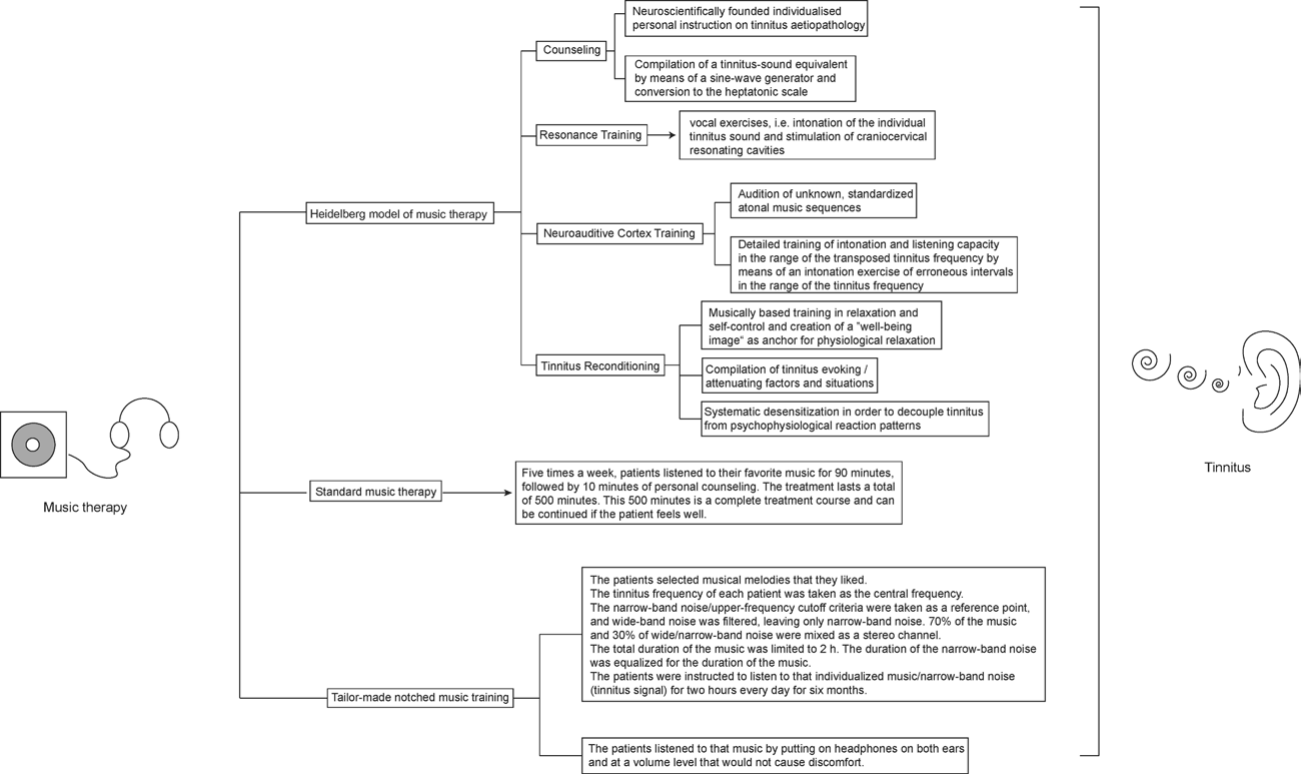
Three types of music therapy.

There are various ways to treat tinnitus, including drug therapy, cognitive behavioral therapy, massage, electrotherapy, habit therapy, tinnitus retraining therapy, music and sound therapy, hearing aids, and so on.^[7]^ However, no universal therapy can significantly and profoundly relieve tinnitus. Most available approaches are based on management strategies to relieve tinnitus distress.^[8,9]^ Music therapy was first proposed to treat tinnitus in 1988, and subsequent studies showed that it alleviates tinnitus primarily by distracting patients.^[10–12]^ Current music therapy mainly includes Heidelberg, standard, and tailor-made notched music therapy (Fig. 1).^[13–15]^ However, the different outcome of these therapies leads to the uncertainty of music therapy in treating tinnitus. Although some articles conducted clinical experiments on tinnitus patients with different music therapies, the results did not show the effectiveness and advantages of music therapy.^[16–18]^ The observed disparities may stem from variations in the employed evaluation methodologies. Nonetheless, owing to the divergent outcomes presented in distinct articles, music therapy effectiveness in addressing tinnitus remains a topic of contentious discussion.

This article aims to summarize the 3 commonly used music therapies and comprehensively analyze their treatment for tinnitus. Specifically, our goal is to address the following questions:

1. Can music therapy relieve tinnitus symptoms?
2. How does music therapy relieve tinnitus symptoms?
3. Which tinnitus symptoms does music therapy mainly relieve?
4. Is there a difference in the efficacy of the 3 kinds of music therapy for tinnitus?
5. How should tinnitus patients choose their music therapy?

We hope to enhance the understanding of music therapy role in tinnitus treatment by addressing the abovementioned questions. We expect this study to raise awareness of music therapy and offer valuable insights for the subsequent management of tinnitus.

## 2. Methods

We followed The Preferred Reporting Items for Systematic Reviews and the PRISMA reporting guidelines to abstract data and conducted this meta-analysis. This systematic review and meta-analysis was not registered in the International Prospective Register of Systematic Reviews database.

### 2.1. Search strategy

We searched PubMed, Cochrane Library, EMBASE, Web of Science, and MEDLINE to obtain potential studies from their inception to May 2023 in all languages. We used the following terms: music therapy and tinnitus. We used Boolean operators “OR” and “AND” to combine the literature searches (Appendix 1, http://links.lww.com/MD/K766). We carefully screened the reference lists by hand-searching to avoid omitting any potential studies. The search was not limited to any language or reference type.

### 2.2. Study selection

All received articles were extracted to Endnote reference management software. Duplicate articles were identified and deleted. Two independent researchers (YLN and YJY) screened titles and abstracts to select potentially eligible publications. Any dispute was settled by negotiation between 2 reviewers (YLN and YJY). If the negotiation fails, a more authoritative expert will participate in further evaluation.

The final criteria for inclusion were as follows:

1. Participants: Patients with tinnitus (aged ≥ 18 years).
2. Intervention: During the treatment, patients received any form of music intervention, including Heidelberg, standard, and tailor-made notched music therapy.
3. Outcomes: Reports have at least one rating scale related to tinnitus.
4. Study design: All randomized and non-randomized controlled trials (RCTs).

The exclusion criteria included conference abstracts, case reports, reviews, letters, biochemical trials, protocols, and studies without relevant outcomes.

### 2.3. Data extraction and quality assessment

Two independent reviewers (YLN and YJY) extracted data and crosschecked it. We prepared a standardized checklist to extract information about the author name, publication year, study characteristics, sample characteristics, intervention measure, intervention time, outcomes, and outcome measurements. If incomplete data were encountered, we tried our best to contact the study authors for complete data or complement the data with statistical techniques.

The Cochrane risk-of-bias tool was used for evaluating the risk of bias in the included RCTs. The methodological index for non-randomized studies was used to evaluate the risk of bias in the included non-RCTs. Any reviewers’ inconsistencies were resolved through discussion and consensus with a third reviewer.

### 2.4. Data analysis

Means and SDs of outcomes were analyzed. All statistical analyses were performed using Stata 12.0. The pooled effect size and 95% CI were reported. Heterogeneity was calculated using *Q* test and I^2^, in which *P* < .1, I^2^ > 50% meant significant heterogeneity. A random-effect model was used when statistically indicated heterogeneity was found. Otherwise, a fixed-effect model is used. If a study used more than one rating scale to assess tinnitus, we analyzed the effect size for measures belonging to the same rating scale. We used the frequency analysis method to compare the efficacy of 3 music therapies. An Egger test was used to investigate the potential publication bias. If there were a limited number of studies (<6), no assessment for publication was conducted.

## 3. Results

### 3.1. Study selection

**Figure 2.**
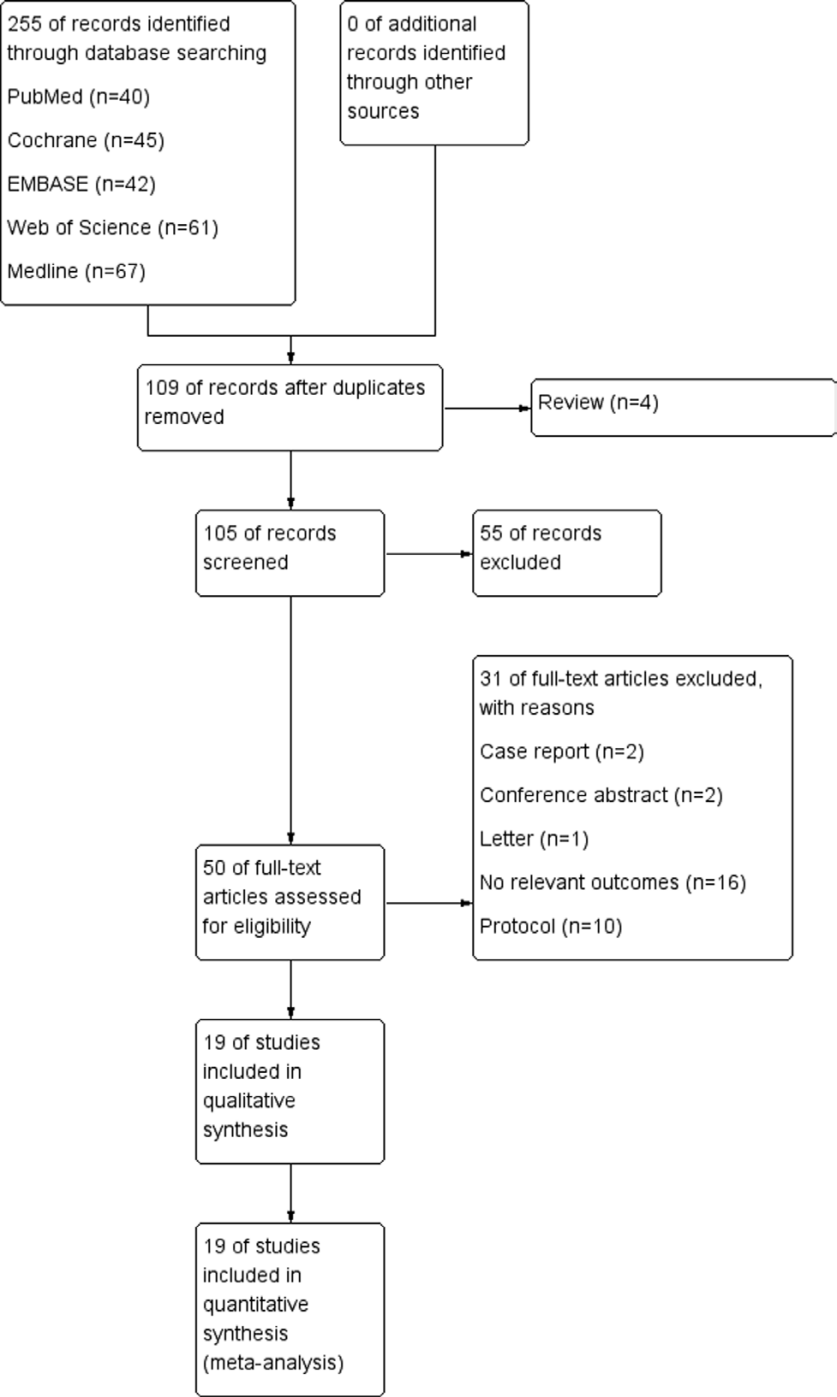
Study flow diagram.

Using the key phrases mentioned above, we identified 255 records from the following databases: 40 from PubMed, 42 from EMBASE, 61 from Web of Science, 45 from Cochrane Library, and 67 from Medline. We remained 105 studies after excluding duplicates and irrelevant references. Then, 55 articles were excluded according to the titles and abstracts. We obtained 50 studies to assess eligibility further. Of these, 19 articles met the inclusion criteria. Finally, 19 articles were eligible and included in the quantitative synthesis. The flow diagram of the study selection process is shown in Figure 2.

### 3.2. Study characteristics

Selected trials included a total of 904 participants who received music therapy, 363 in the Heidelberg model of music therapy (HMOMT) group, 283 in the tailor-made notched music training (TMNMT) group, and 183 in the standard music therapy (SMT) group. These studies included randomized and non-RCTs. All studies reported at least one rating scale related to tinnitus. Information about the characteristics of the included studies was summarized in Table 1.

**Table 1:** Information about the characteristics of the included studies.

Study	Study type	Sample size (M/F)	Age (Y)	Intervention treatment	Number of patients	Duration of treatments	Prior treatment						After treatment						Ref.
							Primary outcomes			Secondary outcomes			Primary outcomes			Secondary outcomes			
							Item	Mean	SD	Item	Mean	SD	Item	Mean	SD	Item	Mean	SD	
Heike et al (2010)	NRCT	14/10	47.7 ± 13.2	HMOMT	24	3 mo	TQ	40.1	11.4	/	/	/	TQ	24	12.2	/	/	/	
Henning et al (2011)	NRCT	/	33.3 ± 4.3	TMNMT	20	1 mo	VAS-ls	56.45	16.04	TQ	7.6	6.3	VAS-ls	55.56	15.78	TQ	6.36	6.14	
Heike et al (2012)	NRCT	65/39	54.3 ± 12.4	HMOMT	104	2.65 ± 1.1 years	TQ	11.9	4.9	/	/	/	TQ	7.4	5.2	/	/	/	
Heike et al (2012)	NRCT	80/39	46.9 ± 16.8	HMOMT	66	6 mo	TQ	39.5	10.6	/	/	/	TQ	24.4	12.2	/	/	/	
Heike et al (2012)	NRCT	80/39	46.9 ± 16.8	SMT	53	6 mo	TQ	41.2	10.5	/	/	/	TQ	22.9	11.5	/	/	/	
Heike et al (2012)	NRCT	80/39	46.9 ± 16.8	MT	119	6 mo	TQ	40.26	10.54	/	/	/	TQ	23.73	11.87	/	/	/	
Newman et al (2012)	NRCT	21/12	55 ± 10.2	TMNMT	33	6 mo	THI	57	15.6	/	/	/	THI	30	15.6	/	/	/	
Grapp et al (2013)	NRCT	12/11	41.3 ± 12.1	HMOMT	23	10 d	THI	13.12	6.17	/	/	/	THI	9.71	5.01	/	/	/	
Heike et al (2015)	NRCT	104/42	45.1 ± 12.4	HMOMT	146	5 d	TQ	31.5	12.2	/	/	/	TQ	17.9	16.5	/	/	/	
Banu et al (2015)	NRCT	7/6	46.8 ± 11.2	MT	13	6 mo	THI	72	21	/	/	/	THI	20	21	/	/	/	
Li et al (2016)	RCT	34/16	55.76 ± 8.47	TMNMT	12	12 mo	THI	44.47	17.2	/	/	/	THI	29.67	15.49	/	/	/	
Li et al (2016)	RCT	34/16	55.76 ± 8.47	SMT	16	12 mo	THI	51.26	20.98	/	/	/	THI	48.13	20.11	/	/	/	
Li et al (2016)	RCT	34/16	55.76 ± 8.47	MT	28	12 mo	THI	48.26	18.74	/	/	/	THI	40.22	22.22	/	/	/	
Kim et al (2016)	NRCT	11/15	51.4 ± 10.6	TMNMT	26	3 mo	THI	33.9	18.9	VAS-ls	5.7	2.2	THI	23.1	15.2	VAS-ls	4.8	2.1	
Hossein et al (2017)	RCT	12/6	53 ± 11	TMNMT	18	3 mo	THI	42.8	21.6	VAS-ls	6.2	2	THI	31.5	20.3	VAS-ls	4.9	1.9	
Nikel et al (2017)	RCT	10/10	51 ± 7	MT	10	6 mo	TQ	46.8	9.6	/	/	/	TQ	24.9	12.83	/	/	/	
Tian et al (2015)	RCT	19/16	47.9 ± 15.4	TMNMT	18	3 mo	THI	54.2	18.7	VAS-ls	6.2	1.5	THI	29.7	16.5	VAS-ls	3.1	1.3	
Tian et al (2015)	RCT	19/16	47.9 ± 15.4	SMT	17	3 mo	THI	44.7	15.7	VAS-ls	5.1	1.7	THI	28.2	17	VAS-ls	4.2	1.9	
Tian et al (2015)	RCT	19/16	47.9 ± 15.4	MT	35	3 mo	THI	49.6	17.7	VAS-ls	5.7	1.7	THI	29	16.5	VAS-ls	3.6	1.7	
Feng et al (2020)	NRCT	12/10	37.41 ± 10.64	MT	22	3 mo	THI	43.3	7.1	/	/	/	THI	40	7.1	/	/	/	
Deniz et al (2020)	NRCT	16/14	44 ± 11	TMNMT	30	6 mo	THI	43.5	21.5	/	/	/	THI	21.8	16	/	/	/	
Diao et al (2021)	RCT	/	47.01 ± 12.71	TMNMT	49	3 mo	THI	44.12	18.09	VAS-ls	4.98	1.49	THI	37	11.33	VAS-ls	3.5	1.07	
Diao et al (2021)	RCT	/	47.01 ± 12.71	SMT	21	3 mo	THI	42.09	13.92	VAS-ls	4.67	1.43	THI	30.5	11.33	VAS-ls	4.1	1.07	
Diao et al (2021)	RCT	/	47.01 ± 12.71	MT	70	3 mo	THI	43.5	16.87	VAS-ls	4.89	1.47	THI	35.02	11.64	VAS-ls	3.68	1.1	
Patorn et al (2021)	RCT	23/26	49.76 ± 12.05	TMNMT	25	3 mo	THI	53.2	16.3	/	/	/	THI	32.7	19.7	/	/	/	
Patorn et al (2021)	RCT	23/26	49.76 ± 12.05	SMT	24	3 mo	THI	52.8	15.4	/	/	/	THI	25	19.3	/	/	/	
Patorn et al (2021)	RCT	23/26	49.76 ± 12.05	MT	49	3 mo	THI	53	15.7	/	/	/	THI	28.93	19.69	/	/	/	
Atipas et al (2021)	RCT	48/56	58 (24–80)	TMNMT	52	6 mo	THI	39.54	23.46	/	/	/	THI	25.76	27.03	/	/	/	
Atipas et al (2021)	RCT	48/56	58 (24–80)	SMT	52	6 mo	THI	38.16	23.46	/	/	/	THI	32.91	27.03	/	/	/	
Atipas et al (2021)	RCT	48/56	58 (24–80)	MT	104	6 mo	THI	38.85	23.35	/	/	/	THI	29.34	27.14	/	/	/	
Sruth et al (2021)	RCT	54/36	47.5 ± 14.7	MT	30	2 mo	THI	2.733	1.014	VAS-ls	4.7	1.784	THI	1.967	1.129	VAS-ls	3.267	1.638	

HMOMT = Heidelberg model of music therapy, M/F = men/female, MT = music therapy, N = number, NRCT = non-randomized controlled study, RCT = randomized controlled trial, Ref = reference, SMT = standard music therapy, THI = tinnitus handicap inventory, TMNMT = tailor-made notched music training, TQ = tinnitus questionnaire, VAS-ls = visual analog scale-loudness score, Y = years.

### 3.3. Risk of bias within studies

**Figure 3.**
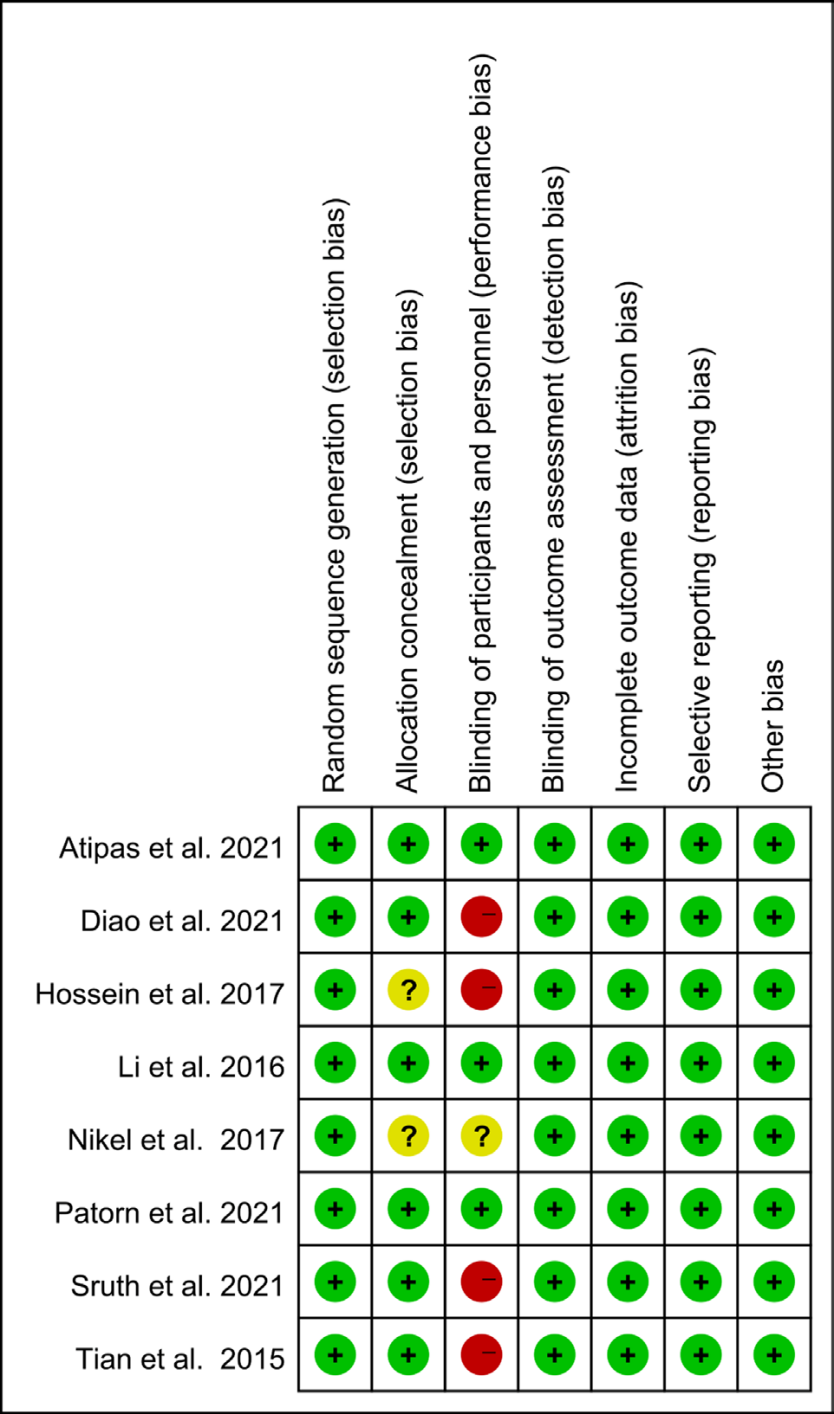
Risk of bias summary of the RCTs. RCTs = randomized controlled trials.

The risk of bias summary of the RCTs was presented in Figure 3. Seven studies fully reported the allocation concealment, and only Hossein et al and Nikel et al did not describe the allocation concealment. All of the RCTs described a methodology of randomization. Blinding of participants is very difficult because of the nature of the intervention. A methodological index for non-randomized studies results for non-RCTs was shown in Table 2. All of these studies were assessed as having a low risk of bias.

**Table 2:** Methodological index for non-randomized studies (MINORS).

Included studies	MINORS												Score
	1	2	3	4	5	6	7	8	9	10	11	12	Total of number of stars
Argstatter et al (2010)	A**	A**	A**	A**	A*	A**	A**	B	B	A**	A**	A**	19
Henning et al (2011)	A**	A*	A**	A**	A*	B	A**	B	A**	A**	A**	A**	18
Heike et al (2012)	A**	A**	A**	A**	A*	A**	A**	B	B	A**	A**	A**	19
Heike et al (2012)	A**	A**	A**	A**	A*	A**	A**	B	A*	B	A**	A**	18
Newman et al (2012)	A**	A**	A**	A**	B	A**	A**	B	A**	B	A**	A**	18
Grapp et al (2013)	A**	A**	A**	A**	B	B	A**	B	B	B	A*	A**	13
Argstatter et al (2015)	A**	A**	A**	A**	A**	A*	A**	B	A**	A**	A**	A**	21
Banu et al (2015)	A**	A**	A**	A**	B	A**	A**	B	B	B	A**	A**	16
Kim et al (2016)	A**	A**	A**	A**	B	A**	A**	B	B	B	A**	A**	16
Feng et al (2020)	A**	A**	A**	A**	B	A**	A**	B	A**	B	A**	A**	18
Deniz et al (2020)	A**	A*	A**	A**	B	A**	A**	B	B	B	A**	A**	15

A*, 1 star; A**, 2 stars; B, no star; 1, A clearly stated aim; 2, Inclusion of consecutive patients; 3, Prospective collection of data; 4, Endpoints appropriate to the aim of the study; 5, Unbiased assessment of the study endpoint; 6, Follow-up period appropriate to the aim of the study; 7, Loss to follow up <5%; 8, Prospective calculation of the study size; 9, An adequate control group; 10, Contemporary groups; 11, Baseline equivalence of groups; 12, Adequate statistical analyses.

### 3.4. Meta-analysis of the effect of music therapy on severity of tinnitus

We evaluated the severity of tinnitus by comparing pre-and post-treatment questionnaire scores. Included studies reported mean differences in tinnitus questionnaire (TQ) or tinnitus handicap inventory (THI).

**Figure 4.**
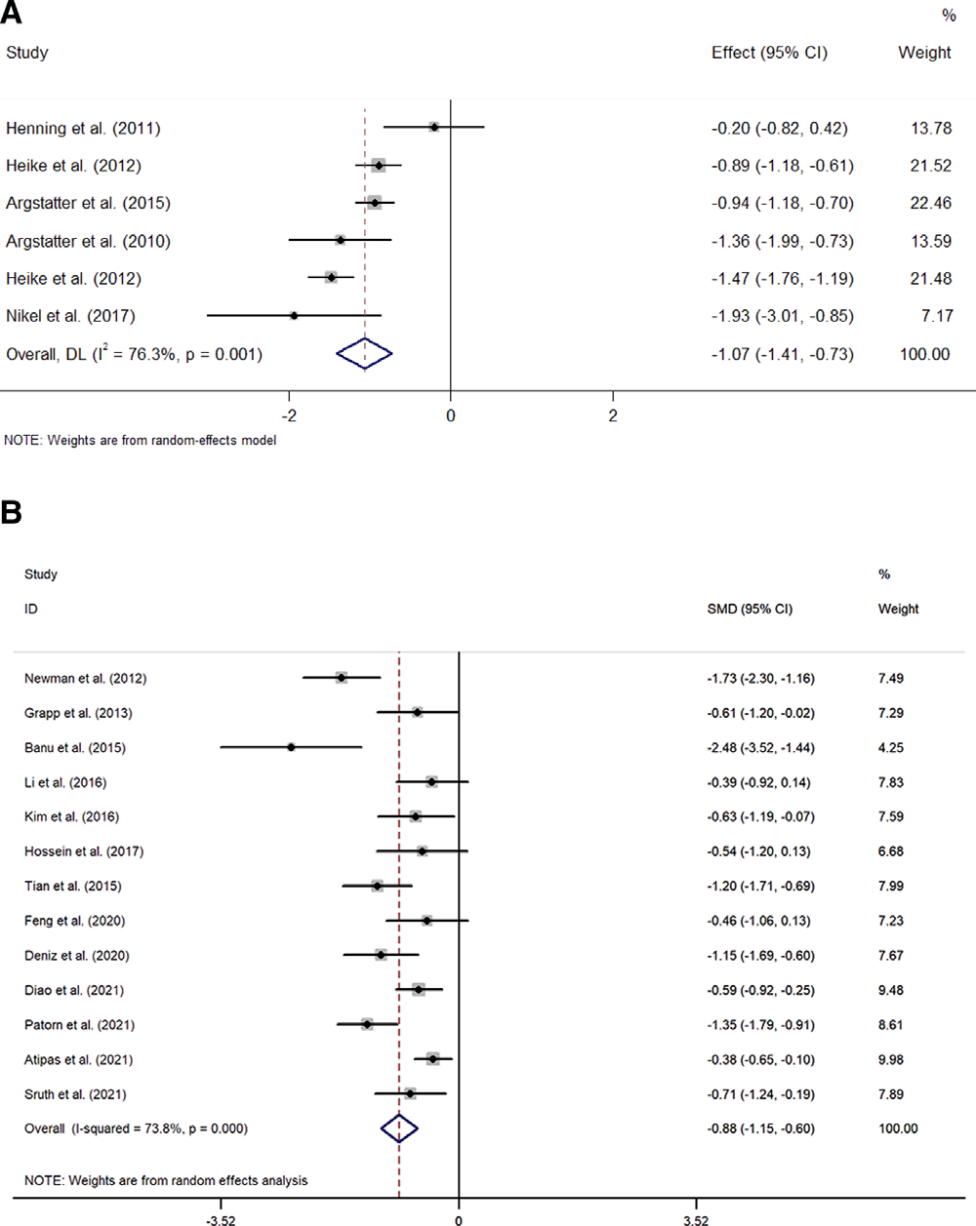
Forest plot of comparing the tinnitus score of pre-and post-treatment. (A) Forest plot of the effect of music therapy on TQ score; (B) Forest plot of the effect of music therapy on THI score. THI = tinnitus handicap inventory, TQ = tinnitus questionnaire.

**Figure 5.**
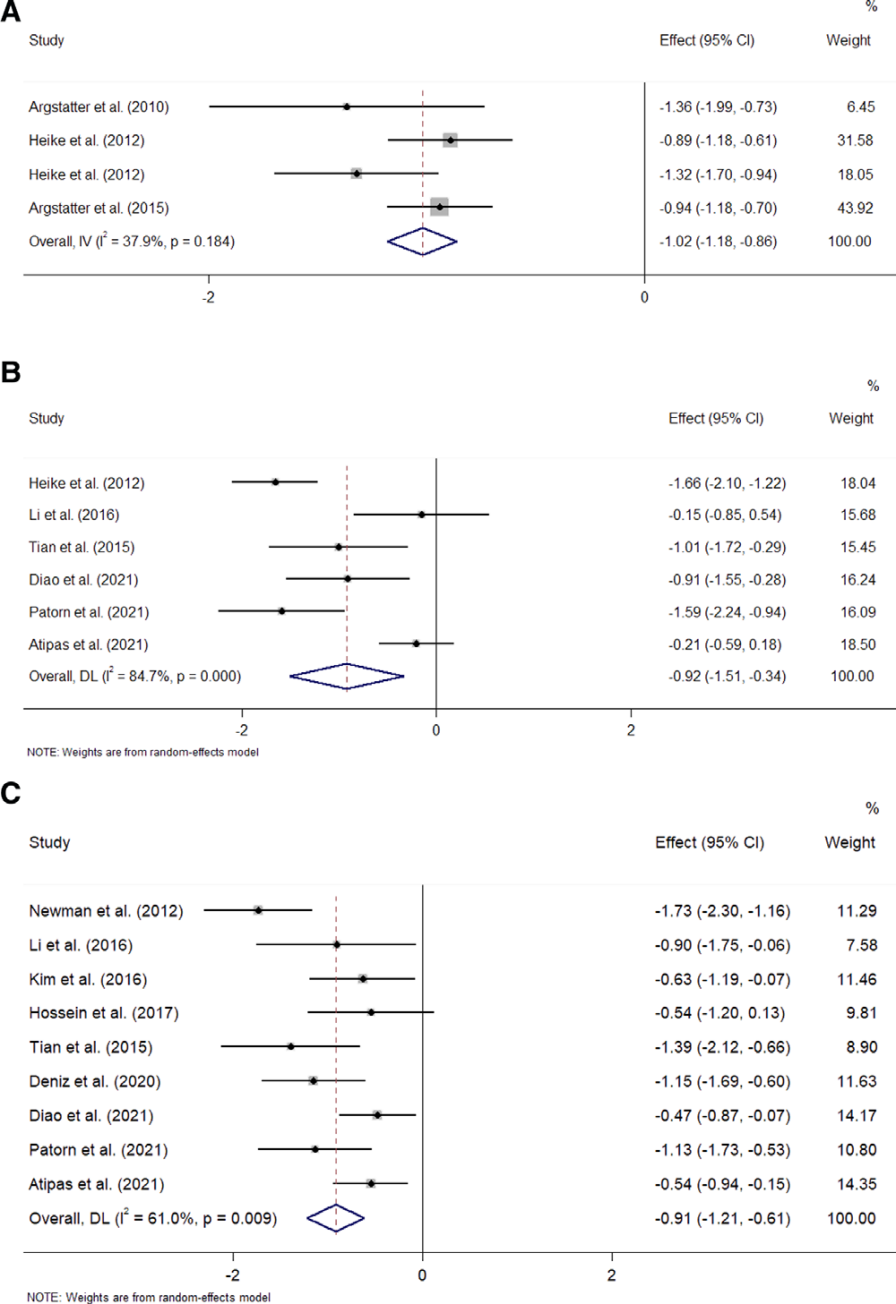
Forest plot of different music therapies: (A) curative effect of HMOMT on tinnitus; (B) curative effect of SMT on tinnitus; and (C) curative effect of TMNMT on tinnitus.

For TQ, the mean difference score between the pre-and post-treatment was available for 6 trials.^[14,17,19–22]^ A random-effect model was used to analyze the data (I^2^ = 76.3% > 50%, *P* = .001 < .1). Compared with before treatment, music therapy significantly reduces the TQ score (SMD = −1.07, 95%CI −1.41 to −0.73, *P* = .004 < .05) (Fig. 4A). Figure 5A is Egger publication bias plot for these results. The plot shows no significant publication bias.

For THI, the mean difference score between the pre-and post-treatment was available for thirteen trials.^[13,15,16,18,23–31]^ A random-effect model was used to analyze the data (I^2^ = 73.8% > 50%, *P* = .000 < .1). Compared with before treatment, music therapy significantly reduces the THI score (SMD = −0.88, 95%CI −1.15 to −0.6, *P* = .000 < .001) (Fig. 4B). Figure 5B is Egger publication bias plot for these results. The plot shows no significant publication bias.

### 3.5. Subgroup analyses

Because of the heterogeneity of music therapy effect on tinnitus, we divided music therapy into 3 subgroups based on different therapeutic methods for meta-analysis and assessed their effects on tinnitus.

#### 3.5.1. Heidelberg model of music therapy.

**Figure 6.**
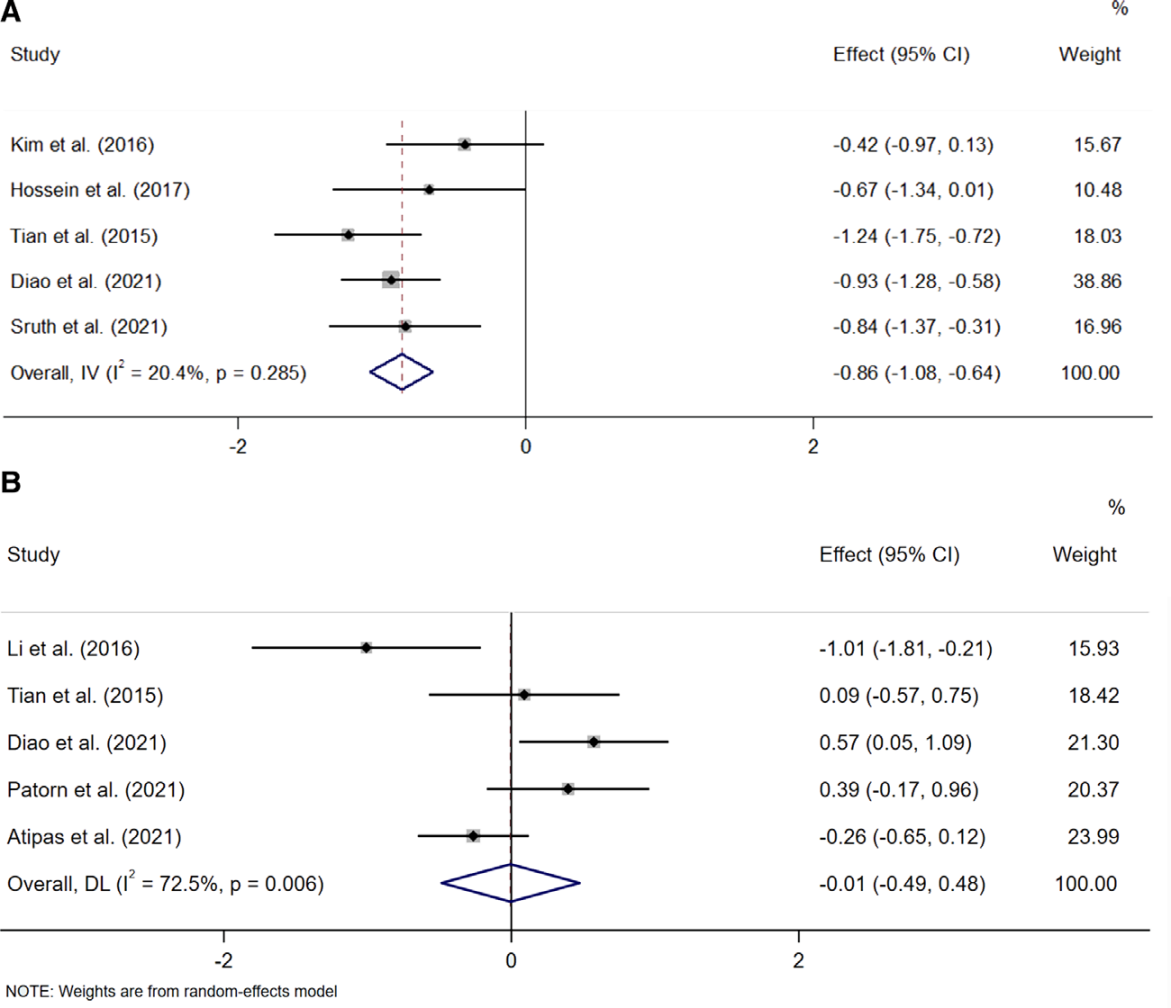
Forest plot of (A) the effect of music therapy on tinnitus loudness and (B) TMNMT vs. SMT.

The HMOMT was reported in 4 articles.^[14,19–21]^ A fixed-effect model was used to analyze the data (I^2^ = 37.9% < 50%, *P* = .184 > .1). HMOMT significantly reduces tinnitus scores. The difference is significant (SMD = −1.02, 95%CI −1.18 to −0.86, *P* = .003 < .05) (Fig. 6A).

Figure 5C is Egger publication bias plot for these results. The plot shows no significant publication bias.

#### 3.5.2. Standard music therapy.

The SMT was reported in 6 articles.^[14–16,28,30,31]^ A random-effect model was used to analyze the data (I^2^ = 84.7% > 50%, *P* = .000 < .1). SMT significantly decreases tinnitus scores. The difference is significant (SMD = −0.92, 95%CI −1.51 to −0.34, *P* = .02 < .05) (Fig. 6B).

Figure 5D is Egger publication bias plot for these results. The plot shows no significant publication bias.

#### 3.5.3. Tailor-made notched music training.

The TMNMT was reported in 9 articles.^[15,16,23,26–31]^ A random-effect model was used to analyze the data (I^2^ = 61% > 50%, *P* = .009 < .1). TMNMT significantly decreases tinnitus scores. The difference is significant (SMD = −0.91, 95%CI −1.21 to −0.61, *P* = .000 < .001) (Fig. 6C). Figure 5E is Egger publication bias plot for these results. The plot shows no significant publication bias.

### 3.6. Meta-analysis of the effect of music therapy on tinnitus loudness

**Figure 7.**
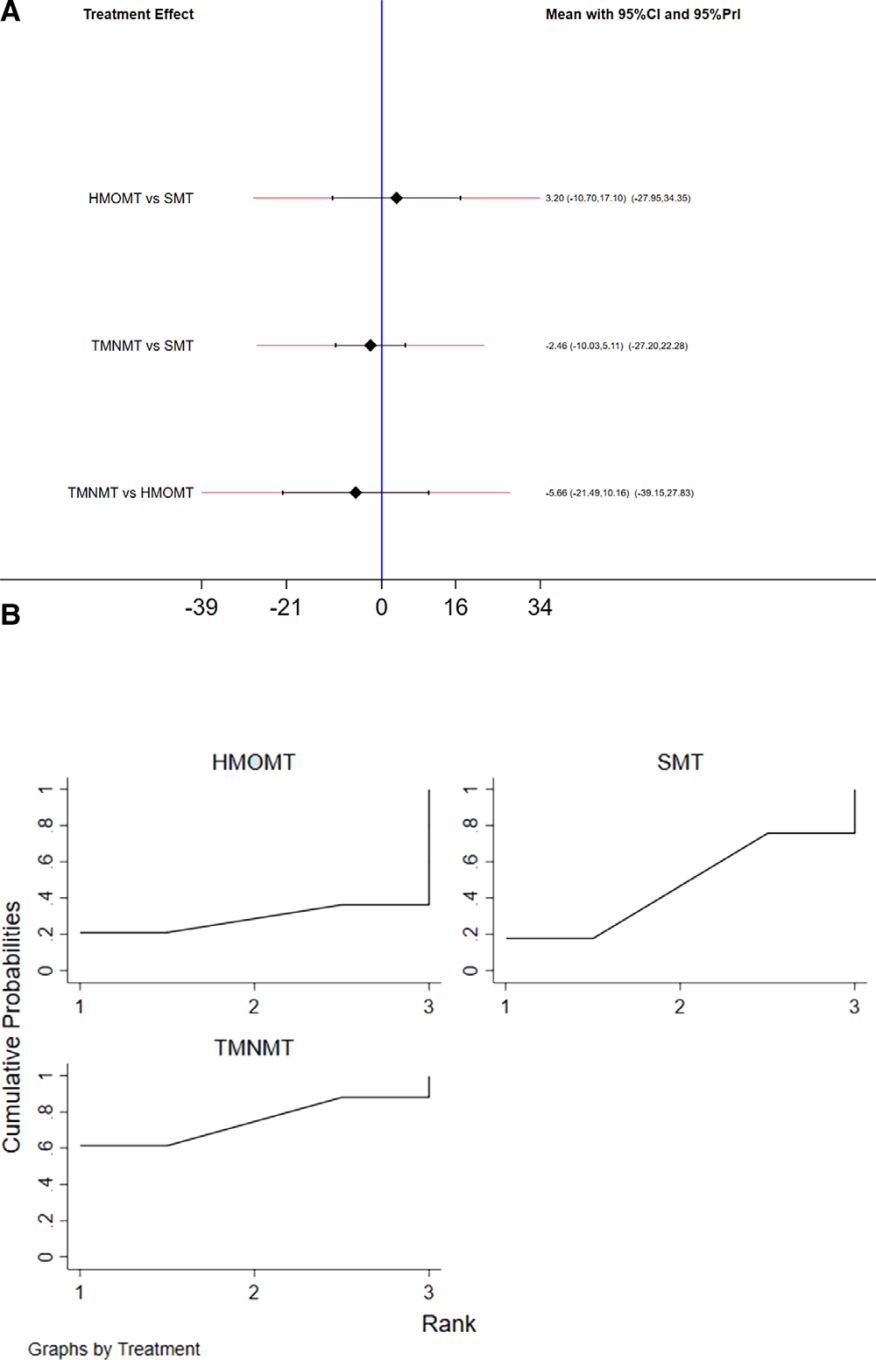
(A) The effect of three music therapies on tinnitus. (B) The order of effectiveness of the three therapies.

We collected visual analog scale score data on loudness to evaluate the degree of tinnitus loudness. Six trials recorded the mean difference score between the pre-and post-treatment.^[13,26–28,30]^ A fixed-effect model was used to analyze the data (I^2^ = 20.4% < 50%, *P* = .285 > .1). We found that music therapy significantly decreases the visual analog scale-loudness score (SMD = −0.86, 95%CI −1.08 to −0.64, *P* = .003 < .05) (Fig. 7A). Figure 5F is Egger publication bias plot for these results. The plot shows no significant publication bias.

### 3.7. Music therapy (TMNMT vs SMT)

Five studies that used THI to measure therapeutic outcomes compared the results of TMNMT and SMT.^[15,16,28,30,31]^ A random-effect model was used to analyze the data (I^2^ = 72.5% > 50%, *P* = .006 < .1). The THI score of the TMNMT group is slightly lower than that SMT group, but the results are not statistically significant (SMD = −0.01, 95%CI −0.49 to 0.48, *P* = .983 > 0.5) (Fig. 7B). Figure 5G is Egger publication bias plot for these results. The plot shows no significant publication bias.

### 3.8. Music therapy (TMNMT vs SMT vs HMOMT)

**Figure 8.**
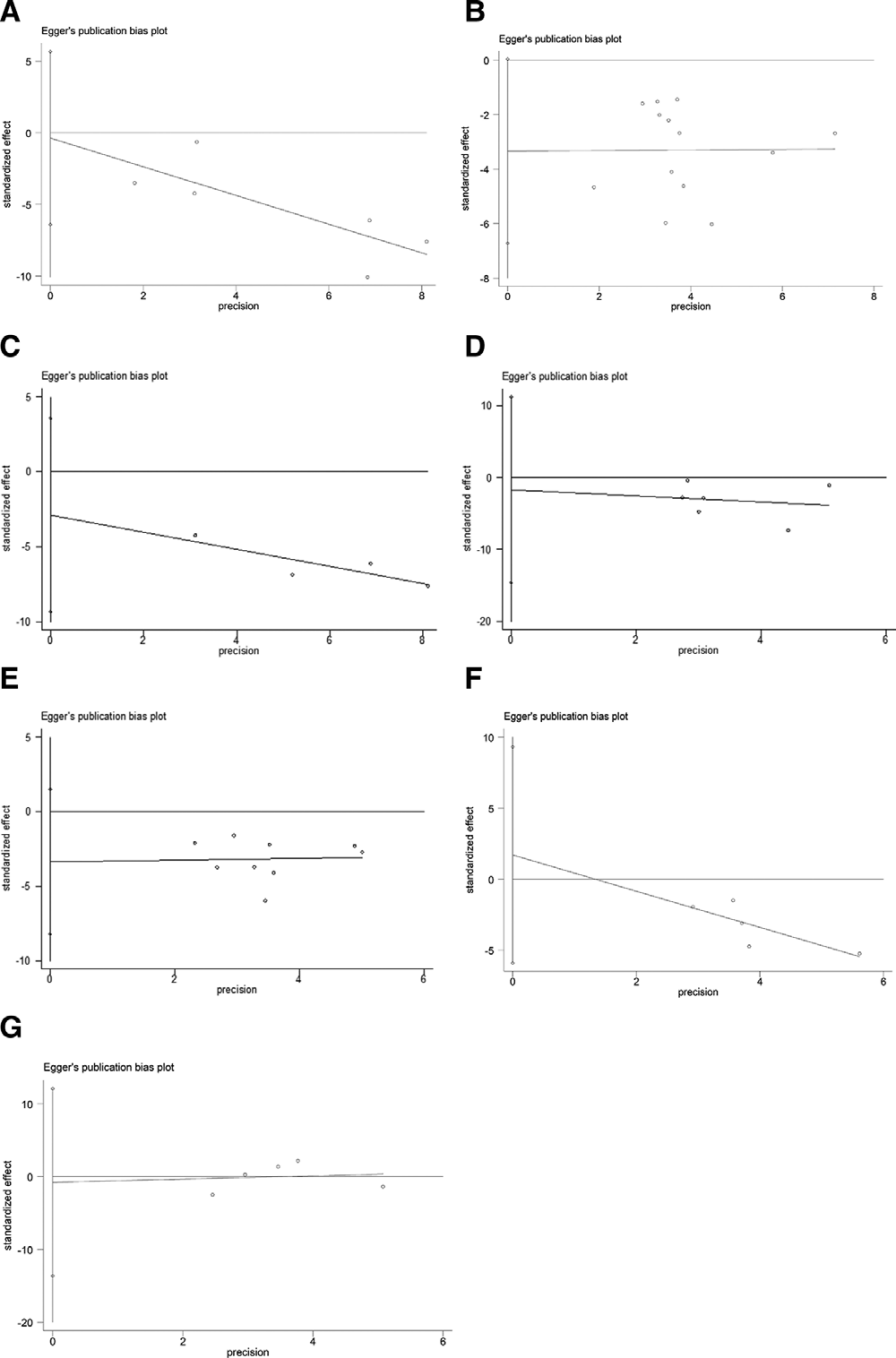
Egger's publication bias plot of (A) music therapy to TQ score, (B) music therapy to THI score, (C) HMOMT, (D) SMT, (E) TMNMT, (F) tinnitus loudness, and (G) TMNMT vs. SMT.

We used the frequency analysis method to compare 3 music therapies. Only 1 article reported comparing HMOMT and SMT, and the relevant research on a direct comparison between HMOMT and TMNMT is missing. We used the data augmentation approach to perform a network meta-analysis. According to the above reasons, forming a complete closed loop for network MATE analysis is difficult. Therefore, we only executed the consistency model here. The result and forest funnel show that TMNMT reduces tinnitus scores more than SMT and HMOMT, and SMT reduces tinnitus scores more than HMOMT (Fig. 8A). Although the order of effectiveness of the 3 therapies is TMNMT > SMT > HMOMT, there is no statistical significance (*P* > .5) (Fig. 8B).

### 3.9. Sensitivity analysis

Sensitivity analysis shows that no individual study significantly impacts the overall results.

## 4. Discussion

The present meta-analysis, comprising 19 clinical trials with 904 individuals with tinnitus, reveals that music therapy could significantly reduce tinnitus scores of tinnitus patients. We divided tinnitus scores into TQ and THI groups, which showed a downward trend after music therapy. This conclusion is further evidence that music therapy can be used to treat tinnitus. Upon comparing our results with prior research, we observed a consistent decrease in both TQ and THI scores post-music therapy, aligning with previous studies. Notably, the influential work of Heike and their team primarily explored the effects of Heidelberg music therapy on tinnitus.^[20]^ Their research also compared the efficacy of Heidelberg music therapy against SMT and music therapy alone for tinnitus treatment, revealing that Heidelberg music therapy significantly alleviated tinnitus symptoms.^[14,19]^ In addition, Nikel et al mainly studied the therapeutic effect of music therapy on chronic diseases, and it is valuable to include the treatment control of chronic tinnitus. Their results suggest that music therapy can indeed treat chronic tinnitus. However, it is essential to acknowledge some disparities between our results and theirs. Our study extracted results from various forms of music therapy used in previous research and conducted a comprehensive analysis with a substantial dataset. Significantly, our study did not limit itself to a single type of music therapy. Our data included any form of music therapy involving tinnitus patients in the treatment process. More precisely, our results demonstrate that tinnitus patients exposed to music as a medium during treatment can reduce tinnitus symptoms. Of course, our study also has some limitations. One notable concern is the variability in patient inclusion criteria across different research studies within music therapy for tinnitus. This variation in criteria introduces the potential for bias when analyzing overall data. While our heterogeneity analysis did not reveal significant differences, it is essential to conduct more extensive studies with standardized inclusion criteria to ensure the robustness of conclusions. Therefore, future research could consider the following approaches:

1. Establishing uniform inclusion criteria, such as accurate diagnosis criteria for chronic tinnitus patients, as well as primary conditions of tinnitus patients, such as age, gender, and duration of disease.
2. The refinement of the research model is a critical consideration. For instance, we recommend implementing a high-confidence, randomized, controlled study design. Under this method, patients undergoing treatment at specific intervals would be randomly allocated to either a control or music therapy group. This approach ensures a robust and impartial assessment of music therapy effectiveness in tinnitus treatment.
3. Extending the treatment and follow-up duration with fixed follow-up assessments at predefined intervals. For instance, a study could involve a 1-year treatment period followed by an evaluation of outcomes.
4. Developing a reliable tinnitus rating scale or incorporating established scales like the TQ and THI into subsequent research evaluations. We recommend the inclusion of these scales to facilitate a comprehensive assessment of tinnitus severity.

Because the tinnitus loudness-related score was collected in our statistical data, we also analyzed the effect of music therapy on tinnitus loudness. We found that music therapy significantly reduces tinnitus-related loudness. This outcome is in line with prior research. In the studies we included, even though tinnitus loudness was just one of the symptoms on the assessment scale, all the data about this aspect indicated a significant reduction in tinnitus loudness following music therapy.^[17,22,26,28,30]^ We believe that music therapy can effectively relieve tinnitus symptoms. However, this only applies to reducing tinnitus symptoms because the data we used to assess the overall efficacy of tinnitus were collected based on subjective tinnitus scores. Some objective indicators, such as audiology measurement, are insufficient for extensive data analysis due to the lack of relevant research.

Since there are different types of music therapy, we conducted a subgroup analysis based on the types of music therapy to exclude the influence of different types on the overall results. Our study divided music therapy into the following 3 types: HMOMT, SMT, and TMNMT. Our results show that all 3 treatments could reduce tinnitus scores. Without a doubt, all 3 types of music therapy are effective in treating tinnitus, a finding consistent with existing research. However, there is ongoing debate regarding which music therapy approach is more effective and can be routinely used to treat tinnitus patients. For this reason, numerous scholars have conducted comparative studies, but a convincing answer has not been reached. For example, the clinical trials of Atipas and Li et al showed that TMNMT was more effective than SMT in improving tinnitus symptoms.^[16,31]^ Conversely, Tian, Diao, and Patorn et al thought there was no significant difference. In order to resolve this controversy, we summarized the results of the current 2 schemes and conducted a meta-analysis. Finally, our results show no significant difference between the 2 schemes in improving tinnitus. Of course, we do not intend to conclude that there is no difference in efficacy between TMNMT and SMT solely based on our results. We still hope to provide patients with an additional opportunity for tinnitus treatment. Therefore, we have carefully reviewed the papers by Atipas and Li, among others, once again. We have identified several issues worthy of attention in their work, which we will outline below for the benefit of future researchers in order to enhance the credibility of subsequent studies:

1. There is a significant loss of patients, resulting in a substantial deviation in the final number of patients analyzed compared to the initial enrollment. In a study like Atipas et al, nearly half of the patients could not remain for data analysis. Similarly, in the study by Li et al, although initially 107 individuals were enrolled, only 34 remained for the actual data analysis at the end. The high patient attrition rate in real-world research introduces a substantial margin of error, making it challenging to have confidence in the final data. Therefore, we suggest that future researchers continue to conduct relevant controlled studies with expanded sample sizes while carefully monitoring patient attrition during the follow-up period and trying to retain patients. If there are instances of patient attrition, it is essential to document the reasons for such attrition, including cases where patients drop out due to poor treatment efficacy, which should be included in the statistics for treatment ineffectiveness. This aspect deserves significant attention for future researchers to draw convincing conclusions.
2. Patient treatment measures need to be standardized, while other factors that may influence treatment outcomes should be excluded. For instance, the study by Atipas et al was well-executed, with patients undergoing only music-related treatments. However, in the study by Li et al, some patients also received medication with undisclosed details about the composition and type of medications, leading to substantial individual differences. We recommend that in future study designs, researchers clearly define the intervention measures for patients and exclude other potential sources of interference. This approach undoubtedly enhances the credibility of the data.

While our results indicate no significant difference in the efficacy of TMNMT and SMT for tinnitus, we recommend that future researchers continue to conduct comparative studies in this area. Music therapy does show effectiveness in tinnitus patients, and the primary significance of conducting such research lies in exploring new and improved music therapy approaches that could offer patients novel avenues for enhanced treatment outcomes.

Moreover, we further analyzed the results of the 3 music therapies. Although TMNMT is superior to SMT and SMT was superior to HMOMT in terms of efficacy ranking, the results are not statistically significant. Thus, in the current situation, there is no apparent difference between the 3 methods in the outcome of tinnitus treatment. We think the leading reason is that they both provide the sound as a desensitization therapy to change the patient response to tinnitus primarily by providing relief from tinnitus prevention.^[32–34]^ As TMNMT is a customized therapy, its treatment process requires high patient cooperation and cost. On the other hand, TMNMT is convenient for treatment because there are no special requirements on the time and place of treatment during the whole treatment process.^[26,27,31]^ The HMOMT integrates tinnitus psychological activation management strategy with special tonal vocalization training to form a fixed and standardized tinnitus treatment training method that must be carried out in professional medical institutions.^[20]^ As for Music therapy, it has the advantage of low cost and high convenience because it only requires regular listening to music.^[31]^ Based on these considerations, it is feasible for tinnitus patients to choose SMT first. In some cases, TMNMT and HMOMT can be used as an alternative to SMT, such as poor psychological quality and high requirements for personal treatment experience. Our results apply only to short-term outcomes after music therapy; long-term follow-up results have yet to be analyzed or discussed. Until now, there have been limited papers that have conducted simultaneous comparative research on all 3 therapies. We found that only the study by Heike et al met the criteria, and their research indicated that HMOMT has an immediate and long-lasting effect.^[14]^ Conducting comparative studies among the 3 music therapies is a research direction that deserves significant attention. To arrive at a convincing conclusion to determine which music therapy is the most effective, researchers must conduct more comparative studies, particularly with large sample sizes, RCTs, and multi-center designs.

In summary, our research results provide valuable insights for further understanding the impact of music therapy on tinnitus. Based on our findings, music therapy can be used as a treatment for tinnitus, particularly for addressing the symptoms of loudness. As for the 3 types of therapy—SMT, TMNMT, and HMOMT—we remain reserved regarding which one has a superior effect on tinnitus. The differences among them in tinnitus treatment are not particularly evident. However, considering the economic cost, time cost, and patient compliance associated with these therapies, if patients are willing to undergo music therapy for tinnitus, we recommend considering SMT as a first option due to its affordability, convenience, minimal side effects, and greater acceptance by patients. Of course, with technological advancements, TMNMT may discover a method to enhance its effectiveness in treating tinnitus by suppressing cortical frequencies. Hence, the research on TMNMT should be supported. With the help of cutting-edge technological advancements, TMNMT therapy can achieve high-quality tinnitus treatment results in the future. Additionally, if a patient has previously failed to achieve success with other music-based methods in treating tinnitus and still wishes to undergo music therapy, considering HMOMT as an alternative is reasonable.

## 5. Limitation

Our study has several limitations. First, the main research objects of the included articles are patients with chronic tinnitus, while there are not many relevant studies on tinnitus caused by other causes. In such situations, music therapy is not practical for all causes of tinnitus because the efficacy of music therapy for tinnitus caused by other causes is unclear. Second, the sample size of each trial is too small. Only 4 studies have a sample size between 100 and 150. Sample sizes for other studies are fewer than 100 patients in all included studies. Although we did not find an association of sample size with the primary outcome, the study size may still be a limitation of this meta-analysis. Third, our meta-analysis included randomized controlled studies and non-randomized controlled studies. Although the patients we finally included were the same group of people before and after the treatment, the RCT is more convincing from the level of clinical evidence. In the future, we look forward to a new, large RCT that further validates the efficacy of music therapy for tinnitus. Fourth, the included trials do not have long-term follow-up results of music therapy for tinnitus, which makes the long-term efficacy of music therapy and recurrence after treatment unclear.

## 6. Conclusion

This meta-analysis of accumulated clinical trial data suggests that music therapy can relieve tinnitus symptoms and loudness. Among music therapies, SMT is recommended first for tinnitus, while TMNMT and HMOMT can be used as alternative therapies for specific cases. However, our recommendations are based on cost, efficacy, and convenience. Comparing the 3 music therapies in tinnitus treatment requires more data and revolutionary research. In addition, the long-term efficacy of music therapy needs further research. We hope that long-term follow-up can be added in future studies to obtain more objective results on the effect of music therapy on tinnitus.

## Author contributions

**Conceptualization:** Yunlian Niu.

**Data curation:** Yunlian Niu.

**Formal analysis:** Yunlian Niu.

**Funding acquisition:** Yijie You.

**Investigation:** Yijie You.

**Methodology:** Yijie You.

**Project administration:** Yijie You.

**Resources:** Yijie You.

**Software:** Yunlian Niu, Yijie You.

**Supervision:** Yijie You.

**Validation:** Yijie You.

**Visualization:** Yijie You.

**Writing – original draft:** Yunlian Niu.

**Writing – review & editing:** Yunlian Niu, Yijie You.

## Supplementary Material



## References

[1] Jastreboff PJ. Phantom auditory perception (tinnitus): mechanisms of generation and perception. Neurosci Res. 1990;8:221–54. [published Online First: 1990/08/01]. 2175858 10.1016/0168-0102(90)90031-9

[2] Heller AJ. Classification and epidemiology of tinnitus. Otolaryngol Clin North Am. 2003;36:239–48. [published Online First: 2003/07/15]. 12856294 10.1016/s0030-6665(02)00160-3

[3] Baguley D McFerran D Hall D. Tinnitus. Lancet. 2013;382:1600–7. [published Online First: 2013/07/06]. 23827090 10.1016/S0140-6736(13)60142-7

[4] Langguth B Salvi R Elgoyhen AB. Emerging pharmacotherapy of tinnitus. Expert Opin Emerg Drugs. 2009;14:687–702. [published Online First: 2009/08/29]. 19712015 10.1517/14728210903206975 PMC2832848

[5] Henry JA Griest S Thielman E. Tinnitus functional index: development, validation, outcomes research, and clinical application. Hear Res. 2016;334:58–64. [published Online First: 2015/06/16]. 26074306 10.1016/j.heares.2015.06.004

[6] McCormack A Edmondson-Jones M Fortnum H. Investigating the association between tinnitus severity and symptoms of depression and anxiety, while controlling for neuroticism, in a large middle-aged UK population. Int J Audiol. 2015;54:599–604. [published Online First: 2015/03/15]. 25766493 10.3109/14992027.2015.1014577 PMC4673512

[7] Elgoyhen AB Langguth B. Pharmacological approaches to the treatment of tinnitus. Drug Discov Today. 2010;15:300–5. [published Online First: 2009/11/26]. 19931642 10.1016/j.drudis.2009.11.003

[8] Hoare DJ Kowalkowski VL Kang S. Systematic review and meta-analyses of randomized controlled trials examining tinnitus management. Laryngoscope. 2011;121:1555–64. [published Online First: 2011/06/15]. 21671234 10.1002/lary.21825 PMC3477633

[9] Kreuzer PM Vielsmeier V Langguth B. Chronic tinnitus: an interdisciplinary challenge. Dtsch Arztebl Int. 2013;110:278–84. [published Online First: 2013/05/15]. 23671468 10.3238/arztebl.2013.0278 PMC3648891

[10] Al-Jassim AH. The use of Walkman Mini-stereo system as a tinnitus masker. J Laryngol Otol. 1988;102:27–8. [published Online First: 1988/01/01]. 3343558 10.1017/s0022215100103871

[11] Eysel-Gosepath K Gerhards F Schicketanz KH. [Attention diversion in tinnitus therapy comparison of the effects of different treatment methods]. HNO. 2004;52:431–9. [published Online First: 2004/05/13]. 15138649 10.1007/s00106-003-0929-4

[12] Jastreboff PJ. Tinnitus retraining therapy. Prog Brain Res. 2007;166:415–23. [published Online First: 2007/10/25]. 17956806 10.1016/S0079-6123(07)66040-3

[13] Sruthi N Venkataramanujam NC Karthikeyan P. A comparative study of treatment outcomes of music therapy, tinnitus maskers and pharmacotherpy in chronic subjective tinnitus. Indian J Otolaryngol Head Neck Surg. 2021;74:185–9. 35813784 10.1007/s12070-021-02799-z PMC9256873

[14] Argstatter H Grapp M Plinkert PK. Heidelberg neuro-music therapy for chronic-tonal tinnitus-treatment outline and psychometric evaluation. Int Tinnitus J. 2012;17:31–41. 23906825

[15] Piromchai P Srisukhumchai C Kasemsiri P. A three-arm, single-blind, randomized controlled trial examining the effects of notched music therapy, conventional music therapy, and counseling on tinnitus. Otol Neurotol. 2021;42:335–40. 33290360 10.1097/MAO.0000000000002935

[16] Li SA Bao L Chrostowski M. Investigating the effects of a personalized, spectrally altered music-based sound therapy on treating tinnitus: a blinded, randomized controlled trial. Audiol Neurootol. 2016;21:296–304. 27838685 10.1159/000450745

[17] Teismann H Okamoto H Pantev C. Short and intense tailor-made notched music training against tinnitus: the tinnitus frequency matters. PLoS One. 2011;6:e24685. 21935438 10.1371/journal.pone.0024685 PMC3174191

[18] Feng T Wang M Xiong H. Efficacy of an integrative treatment for tinnitus combining music and cognitive-behavioral therapy—assessed with behavioral and EEG data. Front Integr Neurosci. 2020;14:12. 32317943 10.3389/fnint.2020.00012 PMC7155387

[19] Argstatter H Grapp M Hutter E. The effectiveness of neuro-music therapy according to the Heidelberg model compared to a single session of educational counseling as treatment for tinnitus: a controlled trial. J Psychosom Res. 2015;78:285–92. 25224125 10.1016/j.jpsychores.2014.08.012

[20] Argstatter H Krick C Plinkert P. Music therapy for noisiform tinnitus concept development and evaluation. HNO. 2010;58:1085–93. 20809193 10.1007/s00106-010-2113-y

[21] Argstatter H Grapp M Hutter E. Long-term effects of the “Heidelberg Model of Music Therapy” in patients with chronic tinnitus. Int J Clin Exp Med. 2012;5:273–88. 22993646 PMC3443887

[22] Neff P Michels J Meyer M. 10 Hz amplitude modulated sounds induce short-term tinnitus suppression. Front Aging Neurosci. 2017;9:130–1. 28579955 10.3389/fnagi.2017.00130 PMC5437109

[23] Newman CW Sandridge SA. A comparison of benefit and economic value between two sound therapy tinnitus management options. J Am Acad Audiol. 2012;23:126–38. 22353681 10.3766/jaaa.23.2.7

[24] Grapp M Hutter E Argstatter H. Music therapy as an early intervention to prevent chronification of tinnitus. Int J Clin Exp Med. 2013;6:589–93. 23936599 PMC3731192

[25] Müjdeci B Köseoğlu S Özcan I. Effect of music therapy on quality of life in individuals with tinnitus. Marmara Med J. 2015;28:38–44.

[26] Kim SY Chang MY Hong M. Tinnitus therapy using tailor-made notched music delivered via a smartphone application and Ginko combined treatment: a pilot study. Auris Nasus Larynx. 2017;44:528–33. 27979609 10.1016/j.anl.2016.11.003

[27] Mahboubi H Haidar YM Kiumehr S. Customized versus noncustomized sound therapy for treatment of tinnitus: a randomized crossover clinical trial. Ann Otol Rhinol Laryngol. 2017;126:681–7. 28831839 10.1177/0003489417725093

[28] Tian RR Diao MF Tian FJ. Preliminary analysis of the effects of tailor-made notched music therapy on chronic idiopathic tinnitus. Zhonghua Er Bi Yan Hou Tou Jing Wai Ke Za Zhi. 2017;52:343–48. 28558452 10.3760/cma.j.issn.1673-0860.2017.05.005

[29] Deniz H Bayazit YA Sarac ET. Individualized treatment of tinnitus during sleep using combined tinnitus signal and music. ORL J Otorhinolaryngol Relat Spec. 2021;83:35–40. 32966989 10.1159/000509981

[30] Diao M Tian R Tian F. Analysis of the effects and predictors of tailor-made notched music therapy for chronic idiopathic tinnitus patients: our experience with 70 patients. Clin Otolaryngol. 2021;46:630–4. 33340429 10.1111/coa.13694

[31] Therdphaothai J Atipas S Suvansit K. A randomized, controlled trial of notched music therapy for tinnitus patients. J Int Adv Otol. 2021;17:221–7. 34100746 10.5152/iao.2021.9385 PMC9450089

[32] Davies E Donaldson I. Tinnitus, membrane stabilizers and taurine. Practitioner. 1988;232(Pt 2):1139. [published Online First: 1988/10/22]. 3256861

[33] DeBartolo HM Jr. Zinc and diet for tinnitus. Am J Otol. 1989;10:256. [published Online First: 1989/05/01]. 2750874

[34] Dulon D Aran JM Schacht J. Potassium-depolarization induces motility in isolated outer hair cells by an osmotic mechanism. Hear Res. 1988;32:123–9. [published Online First: 1988/02/01]. 3360672 10.1016/0378-5955(88)90084-6

