# Supplementary material for: The effect of music therapy on tinnitus: A systematic review
Source: Medicine (Baltimore). 2023 Dec 15;102(50):e36199. doi: 10.1097/MD.0000000000036199 (PMC10727622; doi:10.1097/MD.0000000000036199)
Supplement: Supplementary file 1 [file medi-102-e36199-s001.pdf]

Pubmed

Search number	Search Details	Results
1	"Tinnitus"[Title/Abstract] OR "Tinnitus,Pulsatile"[Title/Abstract] OR "Tinnitus,Clicking"[Title/Abstract] OR "Tinnitus,Objective"[Title/Abstract] OR "Tinnitus,Subjective"[Title/Abstract]	13,244
2	"music therapy"[Title/Abstract] OR "therapy music"[Title/Abstract]	2,900
3	("Tinnitus"[Title/Abstract] OR "Tinnitus,Pulsatile"[Title/Abstract] OR "Tinnitus,Clicking"[Title/Abstract] OR "Tinnitus,Objective"[Title/Abstract] OR "Tinnitus,Subjective"[Title/Abstract]) AND ("music therapy"[Title/Abstract] OR "therapy music"[Title/Abstract])	40

Embase

Search number	Search Details	Results
#1	'tinnitus':ab,ti OR 'ringing-buzzing-tinnitus':ab,ti OR 'ringingbuzzingtinnitus':ab,ti OR 'tinnitus,tensorpalatiniinduced':ab,ti OR 'tensorpalatiniinducedtinnitus':ab,ti OR 'tinnitus,tensortympaniinduced':ab,ti OR 'tensortympaniinducedtinnitus':ab,ti OR 'pulsatiletinnitus':ab,ti OR 'tinnitus,pulsatile':ab,ti OR 'tinnitus,spontaneousoto-acousticemission':ab,ti OR 'tinnitus,spontaneousotoacousticemission':ab,ti OR 'spontaneousoto-acousticemissiontinnitus':ab,ti OR 'spontaneousotoacousticemissiontinnitus':ab,ti OR 'tinnitus,clicking':ab,ti OR 'clickingtinnitus':ab,ti OR 'leudettinnitus':ab,ti OR 'tinnitus,leudet':ab,ti OR 'leudet tinnitus':ab,ti OR 'tinnitus,leudets':ab,ti OR 'tinnitus,noiseinduced':ab,ti OR 'inducedtinnitus,noise':ab,ti OR 'noiseinducedtinnitus':ab,ti OR 'tinnitus,objective':ab,ti OR 'objectivetinnitus':ab,ti OR 'tinnitus,subjective':ab,ti OR 'subjectivetinnitus':ab,ti OR 'tinnitusofvascularorigin':ab,ti OR 'vascularorigintinnitus':ab,ti OR 'tinnitus,vascularorigin':ab,ti	16686
#2	'music therapy':ab,ti OR 'therapy, music':ab,ti	4138
#3	#1 AND #2	42

Cochrane

Search number	Search Details	Results
#1	(music therapy):ti,ab,kw OR (therapy, Music):ti,ab,kw	3056
#2	(Tinnitus):ti,ab,kw OR (Ringing-Buzzing-Tinnitus):ti,ab,kw OR (RingingBuzzingTinnitus):ti,ab,kw OR (Tinnitus,TensorPalatiniInduced):ti,ab,kw OR (TensorPalatiniInducedTinnitus):ti,ab,kw OR (Tinnitus,TensorTympaniInduced):ti,ab,kw OR (TensorTympaniInducedTinnitus):ti,ab,kw OR (PulsatileTinnitus):ti,ab,kw OR (Tinnitus,Pulsatile):ti,ab,kw OR (Tinnitus,SpontaneousOto-AcousticEmission):ti,ab,kw OR (Tinnitus,SpontaneousOtoAcousticEmission):ti,ab,kw OR (SpontaneousOto-AcousticEmissionTinnitus):ti,ab,kw OR (SpontaneousOtoAcousticEmissionTinnitus):ti,ab,kw OR (Tinnitus,Clicking):ti,ab,kw OR (ClickingTinnitus):ti,ab,kw OR (Tinnitus,Leudet):ti,ab,kw OR (LeudetTinnitus):ti,ab,kw OR (Tinnitus,Leudet's):ti,ab,kw OR (Leudet'sTinnitus):ti,ab,kw OR (Tinnitus,Leudets):ti,ab,kw OR (Tinnitus,NoiseInduced):ti,ab,kw OR (InducedTinnitus,Noise):ti,ab,kw OR (NoiseInducedTinnitus):ti,ab,kw OR (Tinnitus,Objective):ti,ab,kw OR (ObjectiveTinnitus):ti,ab,kw OR (Tinnitus,Subjective):ti,ab,kw OR (SubjectiveTinnitus):ti,ab,kw OR (TinnitusofVascularOrigin):ti,ab,kw OR (VascularOriginTinnitus):ti,ab,kw OR (Tinnitus,VascularOrigin):ti,ab,kw	2578
#3	#1 AND #2	45

Web of Science

Search number	Search Details	Results
1	((AB=(music therapy)) OR AB=(therapy Music))	4003
2	((((((((((((((((((((((((((((((AB=(Tinnitus)) OR AB=(Ringing-Buzzing-Tinnitus)) OR AB=(RingingBuzzingTinnitus)) OR AB=(Tinnitus,TensorPalatiniInduced)) OR AB=(TensorPalatiniInducedTinnitus)) OR AB=(Tinnitus,TensorTympaniInduced)) OR AB=(TensorTympaniInducedTinnitus)) OR AB=(PulsatileTinnitus)) OR AB=(Tinnitus,Pulsatile)) OR AB=(Tinnitus,SpontaneousOto-AcousticEmission)) OR AB=(Tinnitus,SpontaneousOtoAcousticEmission)) OR AB=(SpontaneousOto-AcousticEmissionTinnitus)) OR AB=(SpontaneousOtoAcousticEmissionTinnitus)) OR AB=(Tinnitus,Clicking)) OR AB=(ClickingTinnitus)) OR AB=(Tinnitus,Leudet)) OR AB=(LeudetTinnitus)) OR AB=(Tinnitus,Leudet's)) OR AB=(Leudet'sTinnitus)) OR AB=(Tinnitus,Leudets)) OR AB=(Tinnitus,NoiseInduced)) OR AB=(InducedTinnitus,Noise)) OR AB=(NoiseInducedTinnitus)) OR AB=(Tinnitus,Objective)) OR AB=(ObjectiveTinnitus)) OR AB=(Tinnitus,Subjective)) OR AB=(SubjectiveTinnitus)) OR AB=(TinnitusofVascularOrigin)) OR AB=(VascularOriginTinnitus)) OR AB=(Tinnitus,VascularOrigin))	9116
3	#1 AND #2	61

Medline

Search number	Search Details	Results
1	AB music therapy OR AB therapy, Music	3008
2	AB Tinnitus OR AB Ringing-Buzzing-Tinnitus OR AB RingingBuzzingTinnitus OR AB Tinnitus,TensorPalatiniInduced OR AB TensorPalatiniInducedTinnitus OR AB Tinnitus,TensorTympaniInduced OR AB TensorTympaniInducedTinnitus OR AB PulsatileTinnitus OR AB Tinnitus,Pulsatile OR AB Tinnitus,SpontaneousOto-AcousticEmission OR AB Tinnitus,SpontaneousOtoAcousticEmission OR AB SpontaneousOto-AcousticEmissionTinnitus OR AB SpontaneousOtoAcousticEmissionTinnitus OR AB Tinnitus,Clicking OR AB ClickingTinnitus OR AB Tinnitus,Leudet OR AB LeudetTinnitus OR AB Tinnitus,Leudet's OR AB Leudet'sTinnitus OR AB Tinnitus,Leudets OR AB Tinnitus,NoiseInduced OR AB InducedTinnitus,Noise OR AB NoiseInducedTinnitus OR AB Tinnitus,Objective OR AB ObjectiveTinnitus OR AB Tinnitus,Subjective OR AB SubjectiveTinnitus OR AB TinnitusofVascularOrigin OR AB VascularOriginTinnitus OR AB Tinnitus,VascularOrigin	11602
3	(AB Tinnitus OR AB Ringing-Buzzing-Tinnitus OR AB RingingBuzzingTinnitus OR AB Tinnitus,TensorPalatiniInduced OR AB TensorPalatiniInducedTinnitus OR AB Tinnitus,TensorTympaniInduced OR AB TensorTympaniInducedTinnitus OR AB PulsatileTinnitus OR AB Tinnitus,Pulsatile OR AB Tinnitus,SpontaneousOto-AcousticEmission OR AB Tinnitus,SpontaneousOtoAcousticEmission OR AB SpontaneousOto-AcousticEmissionTinnitus OR AB SpontaneousOtoAcousticEmissionTinnitus OR AB Tinnitus,Clicking OR AB ClickingTinnitus OR AB Tinnitus,Leudet OR AB LeudetTinnitus OR AB Tinnitus,Leudet's OR AB Leudet'sTinnitus OR AB Tinnitus,Leudets OR AB Tinnitus,NoiseInduced OR AB InducedTinnitus,Noise OR AB NoiseInducedTinnitus OR AB Tinnitus,Objective OR AB ObjectiveTinnitus OR AB Tinnitus,Subjective OR AB SubjectiveTinnitus OR AB TinnitusofVascularOrigin OR AB VascularOriginTinnitus OR AB Tinnitus,VascularOrigin) AND (S1 AND S2)	67
